# IL-10 induces the development of immunosuppressive CD14^+^HLA-DR^low/−^ monocytes in B-cell non-Hodgkin lymphoma

**DOI:** 10.1038/bcj.2015.56

**Published:** 2015-07-31

**Authors:** B Xiu, Y Lin, D M Grote, S C Ziesmer, M P Gustafson, M L Maas, Z Zhang, A B Dietz, L F Porrata, A J Novak, A-B Liang, Z-Z Yang, S M Ansell

**Affiliations:** 1 Department of Hematology, Tongji Hospital, Tongji University, Shanghai, China; 2 Division of Hematology and Internal Medicine, Mayo Clinic, Rochester, MN, USA; 3 Division of Transfusion Medicine, Mayo Clinic, Rochester, MN, USA

## Abstract

The biological role of monocytes and macrophages in B-cell non-Hodgkin lymphoma (NHL) is not fully understood. We have previously reported that monocytes from patients with B-cell NHL have an immunosuppressive CD14^+^HLA-DR^low/−^ phenotype that correlates with a poor prognosis. However, the underlying mechanism by which CD14^+^HLA-DR^low/−^ monocytes develop in lymphoma is unknown. In the present study, we found that interleukin (IL)-10, which is increased in the serum of patients with B-cell NHL, induced the development of the CD4^+^HLA-DR^low/−^ population. Using peripheral blood samples from patients with B-cell NHL, we found that absolute numbers of CD14^+^ monocytic cells with an HLA-DR^low/−^ phenotype were higher than healthy controls and correlated with a higher International Prognostic Index score. IL-10 serum levels were elevated in lymphoma patients compared with controls and were associated with increased peripheral monocyte counts. Treatment of monocytes with IL-10 *in vitro* significantly decreased HLA-DR expression and resulted in the expansion of CD14^+^HLA-DR^low/−^ population. We found that lymphoma B cells produce IL-10 and supernatants from cultured lymphoma cells increased the CD14^+^HLA-DR^low/−^ population. Furthermore, we found that IL-10-induced CD14^+^HLA-DR^low/−^ monocytes inhibited the activation and proliferation of T cells. Taken together, these results suggest that elevated IL-10 serum levels contribute to increased numbers of immunosuppressive CD14^+^HLA-DR^low/−^ monocytes in B-cell NHL.

## Introduction

B-cell non-Hodgkin lymphoma (NHL) is a serious and frequently fatal illness. The clinical course of this disease is variable, and the molecular and cellular mechanisms responsible for the clinical heterogeneity of B-cell NHL are largely unknown. However, it is becoming increasingly clear that host immune response has an important role in the disease severity, clinical outcome and response to therapy.^1, 2, 3, 4^ In general, while T cells mediate an immune response that is usually favorable for patient outcome,^5, 6, 7, 8^ a monocyte-mediated immune response correlates with an inferior prognosis in B-cell NHL.^9, 10^ One of the mechanisms responsible for the poor prognosis is that monocytes differentiate into a suppressive cell type that inhibits host antitumor immunity.^11, 12, 13^

We have previously reported that monocytes from peripheral blood of B-cell NHL patients exhibit an immunosuppressive phenotype and lymphoma patients have increased numbers of CD14^+^HLA-DR^low/−^ cells that inhibit host antitumor immunity.^14^ In addition, this subpopulation of monocytes is clinically relevant as increased numbers of CD14^+^HLA-DR^low/−^ monocytes correlate with advanced stage of disease.^14, 15^ These results suggest that the CD14^+^HLA-DR^low/−^ population has an important role in monocyte-mediated systemic suppression in B-cell NHL. However, the underlying mechanism by which CD14^+^HLA-DR^low/−^ monocytes develop in patients with B-cell NHL is unknown.

Interleukin-10 (IL-10) is a pleotropic cytokine produced by various types of cells, including T cells, B cells and monocytes, as well as tumor cells. The main biological function of IL-10 is to limit inflammatory responses and regulate differentiation and proliferation of immune cells such as T cells, B cells, natural killer cells, antigen-presenting cells, mast cells and granulocytes.^16^ In the context of tumors, studies have found that IL-10 has both protumoral and antitumoral effects. For example, IL-10 downregulates proinflammatory cytokine expression and acts as an antitumoral cytokine.^17, 18^ In contrast, IL-10 also suppresses antigen-presenting cells thereby allowing tumor cells to evade immune surveillance mechanisms.^17, 19, 20^ In B-cell NHL, it has been shown that serum levels of IL-10 are elevated and elevated levels are associated with an inferior prognosis.^21, 22, 23^ We therefore wished to determine whether IL-10 has a role in regulating the function of monocytes and in defining their phenotype and function.

In the present study, we measured the absolute counts of monocytes and CD14^+^HLA-DR^low/−^ cells in the peripheral blood of patients with B-cell NHL and assessed the effect of IL-10 on the development of CD14^+^HLA-DR^low/−^ monocytes. Furthermore, we evaluated the phenotype and function of CD14^+^HLA-DR^low/−^ cells, as well as biological and clinical relevance of these cells in patients with B-cell NHL.

## Materials and methods

### Patients and controls

Patients providing written informed consent were eligible for this study if they had a tissue biopsy that on pathological review showed B-cell NHL and adequate peripheral blood to perform the experiments. Peripheral blood from healthy donors providing written informed consent was used as control. The use of human specimens samples for this study was approved by the Institutional Review Board of the Mayo Clinic/Mayo Foundation.

### Reagents and cell lines

All cytokines were purchased from PeproTech (Rocky Hill, NJ, USA): macrophages colony-stimulating factor (M-CSF; 50 ng/ml), granulocyte macrophages (GM)-CSF (50 ng/ml), IL-10 (0.1–100 ng/ml), interferon (IFN)-γ (50 ng/ml) and IL-4 (50 ng/ml). The fluorochrome-conjugated antibodies (Abs) for surface staining (CD4, CD14, CD16, CD25, CD32, CD40, CD64, CD69, CD80, CD86, CD142, CD163, CD206, TNFR2, PD-1, B7-H1, HLA-DR) were obtained from BD Pharmingen (San Diego, CA, USA). Antibodies IL-10 receptor α (IL-10Rα), IL-10Rβ and isotype control were purchased from R&D Systems, Minneapolis, MN, USA. The 5-(and-6)-carboxyfluorescein diacetate succinimidyl ester (CFSE) was obtained from Molecular Probes (Eugene, OR, USA). CD4^+^ and CD14^+^ Cell Isolation Kits were purchased from STEMCELL (Vancouver, BC, Canada). B-cell line SuDHL-2 was purchased from German Resource Centre for Biological Material (DSMZ, Braunschweig, Germany) and has been recently tested for mycoplasma contamination as negative.

### Immunophenotyping of peripheral blood

Leukocytes were analyzed by direct Ab staining of whole blood and analyzed by flow cytometry as previously described.^14, 24^ Data were acquired on a Gallios Flow Cytometer (Beckman Coulter, Chaska, MN, USA) and analyzed with the Kaluza v1.2 software (Beckman Coulter).

### Cell isolation and purification

Peripheral blood mononuclear cells (MNCs) were isolated by Ficoll density gradient centrifugation. CD14^+^ monocytes and CD4^+^ T cells were isolated using positive or negative selection by RoboSep (StemCell Technologies, Vancouver, BC, Canada). Purity, as assessed by CD14 or CD4 staining by flow cytometry, was >95%.

### Monocyte treatment

Monocytes were cultured at 2 × 10^6^ cells/well on a 24-well plate for 24 h in RPMI 1640, supplemented with 10% heat-inactivated fetal calf serum (CSL Biosciences, Parkville, VIC, Australia), 2 mm GlutaMax-1 (Invitrogen, Carlsbad, CA, USA), 100 U/ml penicillin and 100 mg/ml streptomycin. Cells were then treated with the following cytokines alone or in combination for 24 h and collected for phenotype detection. For the IL-10 blockade assay, monocytes were pretreated with blocking Abs against IL-10Rα (R&D Systems) and IL-10Rβ (R&D Systems) or isotype control (R&D Systems) for 1 h. After washing, cells were cultured in the presence of IL-10 or supernatant from lymphoma cells.

### Collection of supernatant from lymphoma cell lines and patient cells

Lymphoma cell lines were seeded into a 24-well plate at 3 × 10^6^/ml in RPMI 1640, supplemented with 10% heat-inactivated fetal calf serum, 2 mm GlutaMax-1, 100 U/ml penicillin and 100 mg/ml streptomycin for 24 h. RPMI medium without cells was put into wells as a control. Supernatants were collected at 24 h. Monocytes were cultured in 500 μl of supernatant and 500 μl of fresh media for 24 h. For patient samples, freshly isolated MNCs were cultured in a flask at 2 × 10^8^/cells in 20 ml RPMI for 24 h. Supernatants were collected and stored at −80 °C for future use. Monocytes were cultured with M-CSF 20 ng/ml in 1700 μl fresh RPMI with 300 μl of supernatant from B-NHL patients for 72 h. After culture, monocytes were collected and analyzed for phenotype.

### CFSE labeling and T-cell proliferation assay

CD4^+^ T cells were washed, counted and resuspended at 1 × 10^7^/ml in phosphate-buffered saline. A stock solution of CFSE (5 mm) was diluted 1:100 with phosphate-buffered saline and added to the cells for a final concentration of 5 μm. After 15 min at room temperature, cells were washed three times with 10 volumes of phosphate-buffered saline containing 10% fetal bovine serum. CFSE-labeled T cells were cultured alone or with monocytes (2:1) with human T-Activator CD3/CD28 beads in a 96-well plate at 37 °C in the presence of 5% CO_2_. Cells were harvested at days 3 and 6, washed and stained with phycoerythrin (PE)-anti-CD3 and allophycocyanin (APC)-anti-CD14 for 30 min at 4 °C. Cells were washed twice and acquired and analyzed on a flow cytometer.

### T-cell activation assay

T cells were cultured alone or with monocytes (2:1) with human T-Activator CD3/CD28 beads in a 96-well plates at 37 °C in the presence of 5% CO_2_. Cells were harvested at day 3. After washing, cells were stained with PE-anti-CD25, Percp-anti-CD69 and APC-anti-CD3 for 30 min at 4 °C and analyzed by flow cytometer.

### Luminex and enzyme-linked immunosorbent assay (ELISA)

IL-10 levels in serum and culture supernatants were measured by Luminex (Invitrogen) or ELISA (R&D Systems), respectively. Serum was collected from NHL patients before treatment. For the Luminex assay, the serum specimens were thawed, clarified by centrifugation and assayed according to the manufacturer's instruction. The specimens were analyzed on a Luminex 200 instrument and results were generated using the STarStation software (Applied Cytometry, Sheffield, UK).

For ELISA, freshly isolated MNCs from lymphoma patients were cultured in the presence of lipopolysaccharide 1 μg/ml for 24 h. Lymphoma cell lines were seeded in a 24-well plate at 3 × 10^6^/ml for 24 h. Supernatants were collected from the culture and assayed with an ELISA Kit (R&D Systems) according to the manufacturer's instructions. The optical density of each well was determined using a SpectraMax 190 microplate reader (Molecular Devices, Sunnyvale, CA, USA) set to 450 nm and analyzed using the SoftMax Pro 5 software (Molecular Devices).

### Statistics

Comparisons among groups were evaluated using Student's *t*-test and Wilcoxon rank-sum tests. Statistical analyses were performed using the Prism 5 and SigmaPlot software, and *P*-values <0.05 were considered statistically significant.

## Results

### Absolute numbers of monocytes increased in B-cell NHL

**Figure 1:**
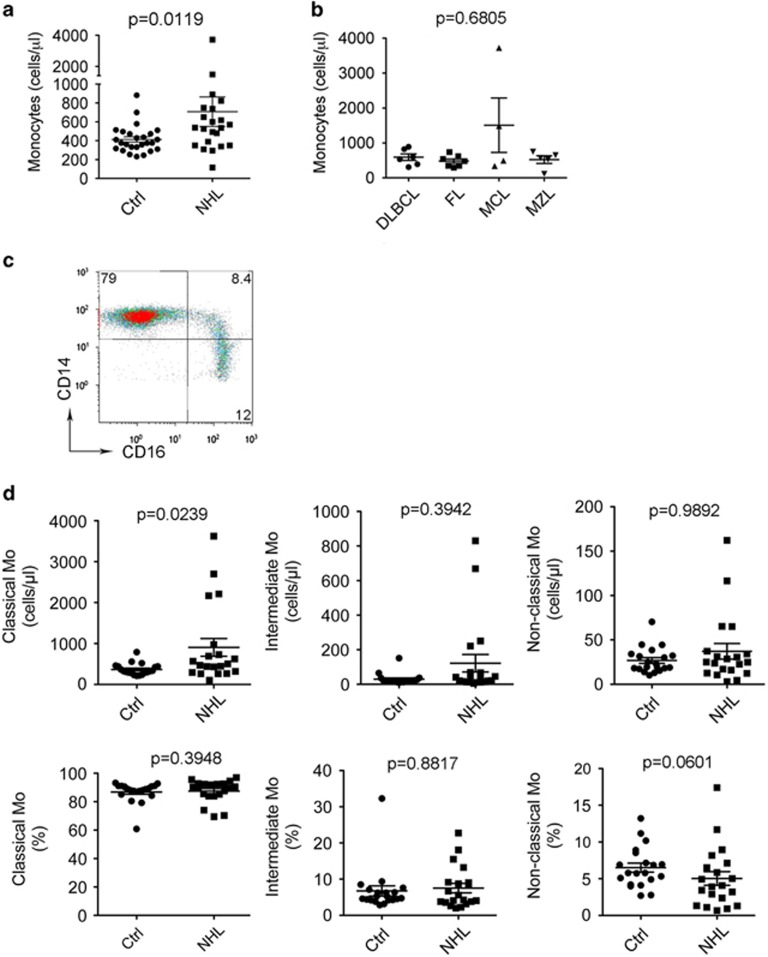
Absolute numbers of monocytes are increased in B-cell NHL. (**a** and **b**) A graph showing absolute monocyte counts in blood from NHL patients and healthy donors (**a**) or patients with different histologies (**b**) measured by flow cytometry. DLBCL, diffuse large B-cell lymphoma; FL, follicular lymphoma; MCL, mantle cell lymphoma; MZL, marginal zone lymphoma. One milliliter freshly drawn whole blood was stained with a panel of Abs and analyzed by flow cytometry. Absolute monocyte counts were calculated as the numbers of CD14^+^ cells per microliter of blood (NHL: *n*=22, Ctrl: *n*=26). (**c**) Representative plots showing coexpression of CD14 and CD16 in blood from a healthy donor. Classical monocytes: CD14^++^CD16^−^; intermediate monocytes: CD14^++^CD16^+^; non-classical monocytes: CD14^+^CD16^++^. (**d**) Graphs showing the absolute counts (upper panel) or percentages (lower panel) of classical, intermediate and non-classical monocytes (Mo) in blood from NHL patients and healthy donors measured by flow cytometry.

To determine the role of immune cells in NHL, we first measured the numbers of mononuclear phagocytes (monocytes, myeloid-derived suppressor cells, macrophages, dendritic cells), T cells and natural killer T cells by flow cytometry in the peripheral blood from 22 newly diagnosed NHL patients, including diffuse large B-cell lymphoma (*n*=6), follicular lymphoma (*n*=7), mantle cell lymphoma (*n*=4) and marginal zone lymphoma (*n*=5), and healthy donors matched for age and gender (*n*=26). As shown in Figure 1a, the absolute numbers of CD14^+^ monocytes were higher in patients with B-cell NHL (707.7±155.4) than healthy donors (414.2±28.32, *P*=0.0119). There was no significant difference, however, in the absolute number of monocytes among different histological types of lymphoma (Figure 1b). There was also no significant difference of absolute numbers of myeloid-derived suppressor cells, T cells and natural killer T cells between lymphoma patients and healthy donors (data not shown).

Human monocytes were then analyzed in three distinct subsets: classical (CD14^++^CD16^−^), intermediate (CD14^++^CD16^+^) and non-classical (CD14^+^CD16^++^) monocytes,^25^ and we measured the absolute numbers of these monocytic subsets by flow cytometry. The gating strategy to identify the three subsets (CD14^++^CD16^−^, CD14^++^CD16^+^ and CD14^+^CD16^++^) is shown in Figure 1c. Although we did not see a significant difference of intermediate or non-classical monocytes between patients and healthy donors (Figure 1d), the absolute numbers of classical monocytes were significantly increased in NHL patients (NHL: 903.4±216.4, Ctrl: 366.3±31.43, *P*=0.0239), probably owing to an increase in absolute numbers of monocytes in NHL patients (Figure 1d, upper panel). In agreement with this finding, we found that the percentage of non-classical monocytes was decreased in NHL patients (5.04±0.93, *n*=20) compared with healthy donors (6.51±0.62, *n*=20, Figure 1d, lower panel).

### Monocytes from B-cell NHL exhibited CD14^+^HLA-DR^low/−^ phenotype

**Figure 2:**
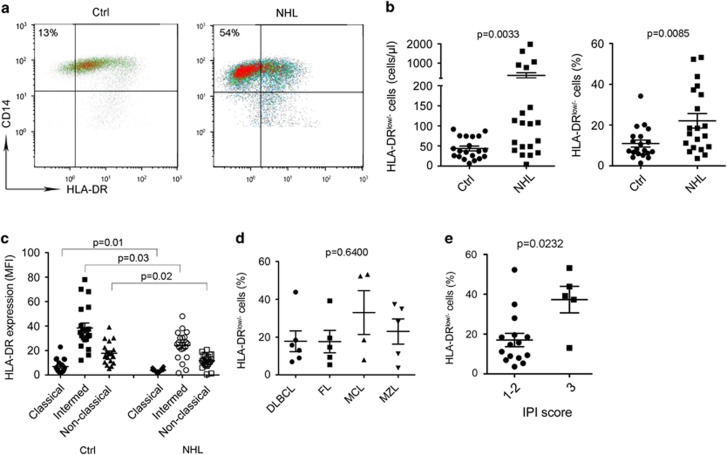
Monocytes from B-cell NHL exhibit CD14^+^HLA-DR^low/−^ phenotype. (**a**) Representative plots showing coexpression of CD14 and HLA-DR in blood from healthy donors (Ctrl) and lymphoma patients (NHL). CD14^+^HLA-DR^low/−^ cells were defined based on isotype control gate. (**b**) Graphs showing absolute counts (left) or the percentage (right) of CD14^+^HLA-DR^low/−^ cells in blood from NHL patients and healthy donors measured by flow cytometry. (**c**) Graphs showing expression level of HLA-DR on classical, intermediate and non-classical monocytes in blood from NHL patients and healthy donors. (**d**) A graph showing the percentage of CD14^+^HLA-DR^low/−^ monocytes in blood from NHL patients with different histologies. (**e**) Graphs showing the percentage of CD14^+^HLA-DR^low/−^ monocytes in blood from NHL patients with different International Prognostic Index (IPI) scores.

To further define the phenotype of peripheral blood monocytes in B-cell NHL patients, we measured the expression of a panel of surface markers. Expression of most surface markers such as CD80, CD86, CD206, CD163, CD40, PD-1, B7-H1, CD169 and CD142 showed no significant difference between patients and healthy donors (data not shown). However, we observed that the surface expression level of HLA-DR on CD14^+^ monocytes was decreased in lymphoma patients compared with healthy donors (Figure 2a), resulting in an increased population of CD14^+^HLA-DR^low/−^ monocytes. Supporting this finding, we found that the absolute numbers and percentages of CD14^+^HLA-DR^low/−^ monocytes were significantly higher in lymphoma patients than in healthy donors (Figure 2b). Furthermore, the increased population of CD14^+^HLA-DR^low/−^ cells came mainly from the classical monocyte population in both lymphoma patients and healthy donors (Figure 2c). We found that there was no significant difference in the percentage of HLA-DR^low^ monocytes among the subtypes of lymphoma, although there seemed to be with a higher percentage of HLA-DR^low^ monocytes in patients with mantle cell lymphoma or marginal zone lymphoma (Figure 2d). Small sample numbers in each histology group may, however, account for this negative finding. Although the numbers of CD14^+^HLA-DR^low/−^ monocytes were not significantly different among the subtypes of lymphoma, patients with a higher International Prognostic Index score had statistically a higher percentage of CD14^+^HLA-DR^low/−^ monocytes (Figure 2e).

**Figure 3:**
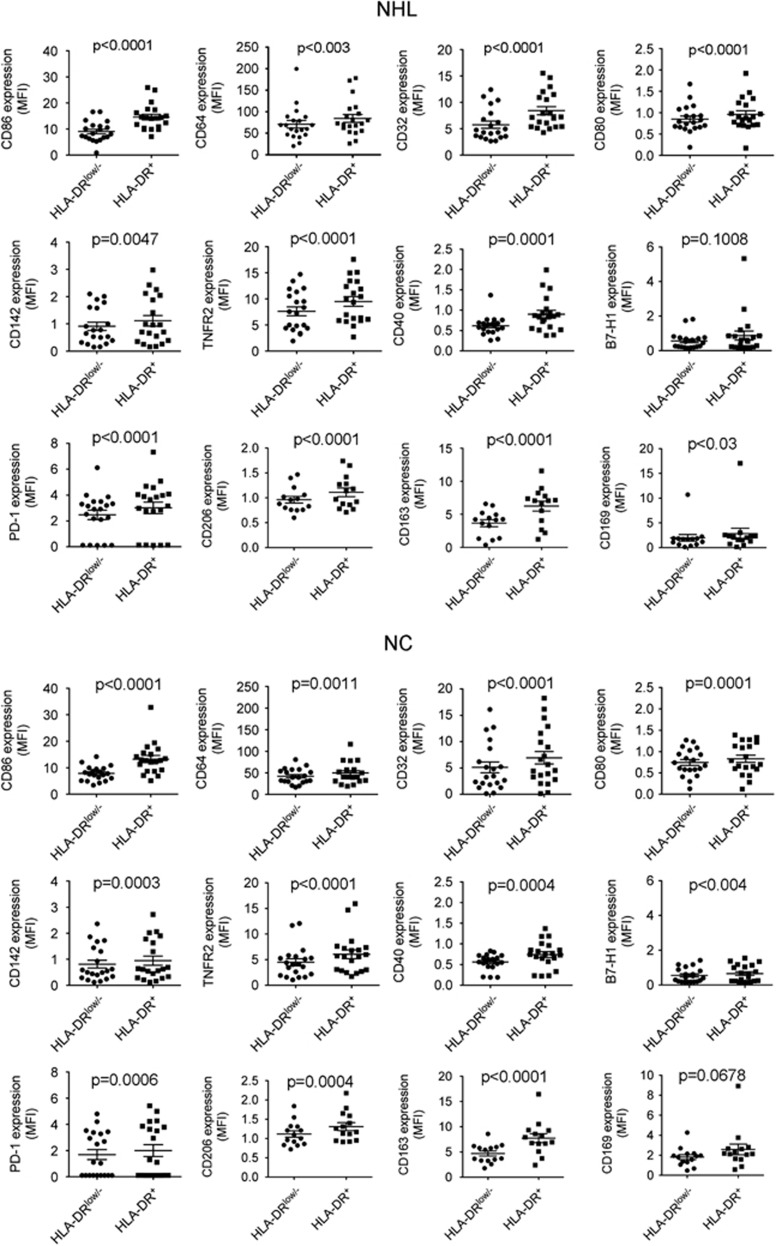
Phenotype of CD14^+^HLA-DR^low/−^ or HLA-DR^+^ monocytes in B-cell NHL. A 10-color flow cytometry was employed to determine the expression of surface markers included CD86, CD64, CD32, CD80, CD142, TNFR2, CD40, B7-H1, PD-1, CD206, CD163 and CD169 on CD14^+^HLA-DR^low/−^ or CD14^+^HLA-DR^+^ monocytes from both healthy donors (NC) or lymphoma patients (NHL).

Next we determined the phenotype of CD14^+^HLA-DR^low/−^ monocytes from B-cell NHL patients by using 10-color flow cytometry. Markers analyzed included CD80, CD86, CD64, CD32, CD163, CD169, CD206, TNFR2, CD40, B7-H1, PD-1, and CD142. As shown in Figure 3, both CD14^+^HLA-DR^+^ and CD14^+^HLA-DR^low/−^ monocytes expressed some of these markers, including CD86 and CD64. However, the expression of these cell surface markers was generally lower in HLA-DR^low/−^ cells compared with HLA-DR^+^ monocytes, although some of these surface markers were expressed at a low level on both HLA-DR^low/−^ and HLA-DR^+^ cells. There was no difference in the profile of cell surface markers when HLA-DR^low/−^ and HLA-DR^+^ populations were compared between lymphoma patients and healthy individuals. We did, however, observe a difference of B7-H1 expression between HLA-DR^+^ and HLA-DR^low^ monocytes in normal controls but not in lymphoma patients based on the *P*-value. However, B7-H1 expression on monocytes was very low in both normal controls and NHL patients. This finding may therefore have limited biological significance. Overall, our findings suggest that HLA-DR^low/−^ monocytes are in fact a physiological population that is expanded in lymphoma patients.

### IL-10 induced the development of CD14^+^HLA-DR^low/−^ monocytes

**Figure 4:**
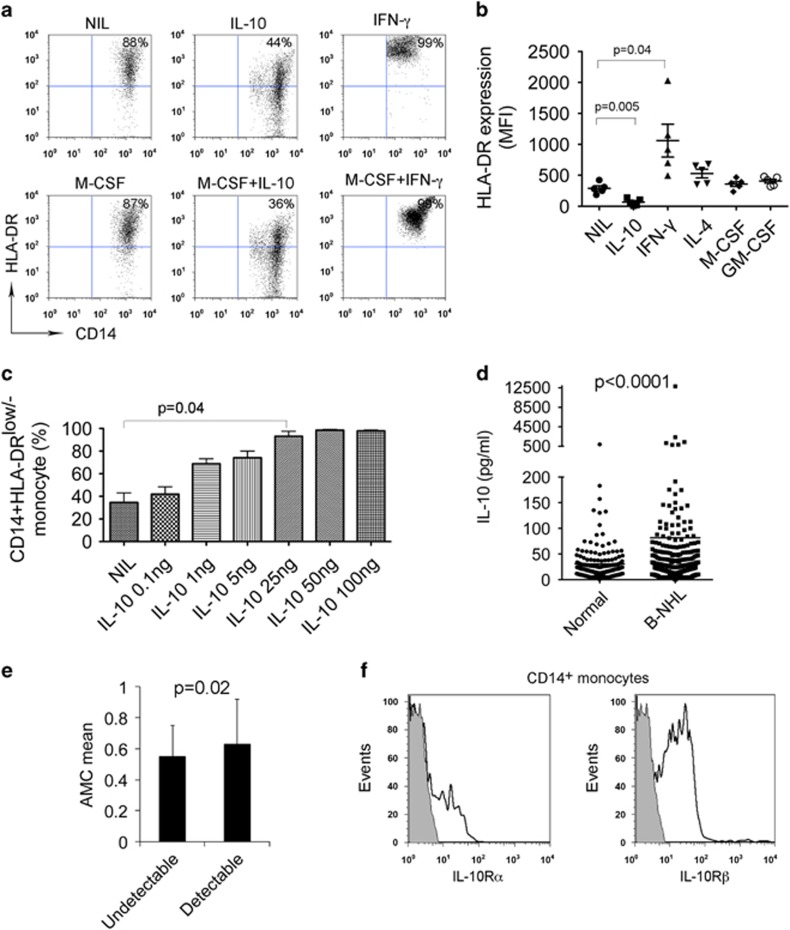
IL-10 induces the development of CD14^+^HLA-DR^low/−^ monocytes. (**a**) Representative plots showing the expression of HLA-DR on CD14^+^ monocytes treated with or without IL-10 or IFN-γ in the presence or absence of M-CSF for 24 h. (**b**) Summarization of HLA-DR expression level in CD14^+^ monocytes treated with or without IL-10, IFN-γ, IL-4, M-CSF or GM-CSF. (**c**) Graph showing the percentage of HLA-DR^low/−^ cells of CD14^+^ monocytes treated with IL-10 at escalating doses. (**d**) Graph showing serum IL-10 level in healthy donors or patients with B-cell NHL. IL-10 concentration was measured by multiplex ELISA (Luminex). (**e**) Graph showing absolute monocyte counts (AMC)/μl in lymphoma patients with undetectable (<20 pg/ml, *n*=118) or detectable (>20 pg/ml, *n*=101) IL-10 serum levels. (**f**) Representative histograms showing the expression of IL-10Rα or IL-10Rβ on CD14^+^ monocytes in patients with B-cell NHL.

To identify which cytokine may be involved in the expansion of CD14^+^HLA-DR^low/−^ monocytes in B-cell NHL, we tested a panel of cytokines for their ability to decrease HLA-DR expression on CD14^+^ monocytes. We treated monocytes from peripheral blood of healthy donors with IL-10, IL-4, IFN-γ, GM-CSF and M-CSF alone or in combination for 24 h and then measured HLA-DR expression. As shown in Figure 4a, the majority of untreated CD14^+^ monocytes had HLA-DR expression on cell surface and the number of HLA-DR^low/−^ cells was low. Treatment of CD14^+^ monocytes with IL-10, however, decreased HLA-DR expression and increased the number of CD14^+^HLA-DR^low/−^ cells. As a control, IFN-γ treatment strongly enhanced the expression of HLA-DR on CD14^+^ monocytes and resulted in a disappearance of CD14^+^HLA-DR^low/−^ cells. Similar treatment with multiple cytokines found that IL-10 remained the only cytokine that significantly downregulated the HLA-DR expression on CD14^+^ monocytes (Figure 4b). We then confirmed that IL-10 induced the development of CD14^+^HLA-DR^low/−^ cells in a dose-dependent manner (Figure 4c).

Next we measured the serum levels of IL-10 from newly diagnosed B-cell NHL patients (diffuse large B-cell lymphoma: *n*=188; follicular lymphoma: *n*=234) and healthy donors (*n*=400) using a multiplex ELISA (Luminex). We found that serum IL-10 levels were significantly elevated in lymphoma patients compared with healthy donors (Figure 4d). Interestingly, we found that patients with detectable serum IL-10 levels had significantly higher absolute numbers of monocytes per microliter than those with undetectable IL-10 levels (Figure 4e). To confirm that monocytes from lymphoma patients are potentially susceptible to the increased IL-10 levels, the expression of IL-10R was assessed via flow cytometry in CD14^+^ monocytes from patients. As shown in Figure 4f, CD14^+^ monocytes expressed both the IL-10Rα and IL-10Rβ subunits of the IL-10R.

### Lymphoma cells produce IL-10 and promote the development of CD14^+^HLA-DR^low/−^ monocytes

**Figure 5:**
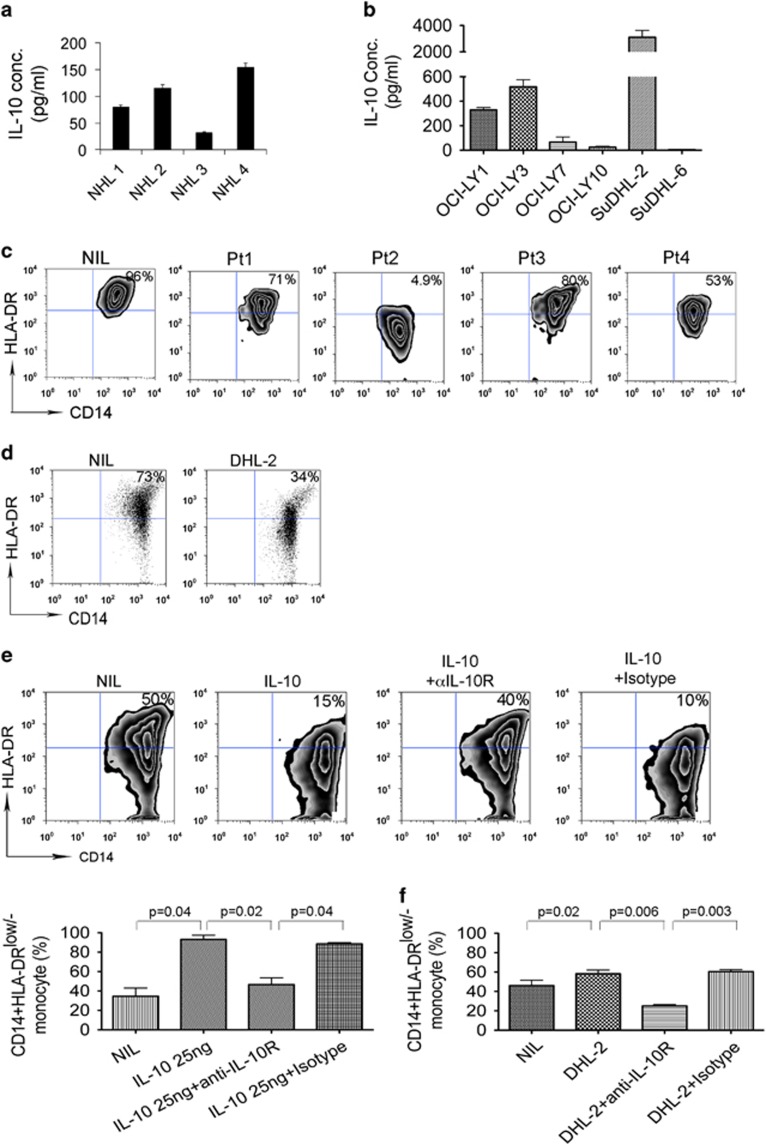
Lymphoma cells produce IL-10 and induce the development of CD14^+^HLA-DR^low/−^ monocytes. (**a** and **b**) Graphs showing IL-10 concentration in culture supernatant of MNCs from lymphoma tissues (**a**) or lymphoma cell lines (**b**). IL-10 concentration was measured by ELISA. (**c** and **d**) Representative plots showing HLA-DR expression on CD14^+^ cells cultured with or without supernatant of lymphoma cells (**c**) or SuDHL-2 cells (**d**). (**e**) Representative plots showing HLA-DR expression on CD14^+^ cells treated with or without IL-10 in the presence of αIL-10R Ab or isotype control. The percentage of CD14^+^HLA-DR^low/−^ cells from above experiment setting was summarized in the graph below (*n*=3). (**f**) Graph showing the percentage of HLA-DR^low/−^ cells in CD14^+^ monocytes cultured with or without supernatant from SuDHL-2 cells in the presence of αIL-10R Ab or isotype control (*n*=3).

Given the elevated IL-10 serum levels in B-cell NHL patients, we then examined the ability of lymphoma cells to produce IL-10. Freshly isolated MNCs from lymphoma biopsy specimens were cultured in the presence of lipopolysaccharide for 24 h. The culture supernatants were collected and IL-10 levels were measured by ELISA. As shown in Figure 5a, lipopolysaccharide-activated MNCs from patient biopsy specimens produced a substantial amount of IL-10. Using lymphoma B-cell lines, we found that lymphoma cells were able to secret IL-10 variably with a very high amount of IL-10 produced by SuDHL-2 cells (Figure 5b). These results suggest that lymphoma B cells produce IL-10, with patient-to-patient variation and cell-line-dependent heterogeneity.

Next we tested the effect of endogenous IL-10 production by lymphoma cells on HLA-DR expression on CD14^+^ monocytes. To do this, we collected supernatants from MNCs of lymphoma tissues or cell lines and cultured CD14^+^ monocytes with these supernatants. We found that the supernatants from lymphoma MNCs downregulated the HLA-DR expression on CD14^+^ monocytes (Figure 5c). Similar results were seen in monocytes cultured with supernatants from SuDHL-2 cells. As shown in Figure 5d, HLA-DR expression was attenuated in CD14^+^ monocytes cultured with supernatants from SuDHL-2 cells.

To determine whether downregulation of HLA-DR expression can be blocked by αIL-10R Ab, we treated CD14^+^ monocytes with IL-10 in the presence of αIL-10Rα (1 μg/ml) plus αIL-10Rβ (10 μg/ml) Ab or isotype control. As shown in Figure 5e, IL-10 25 ng/ml strongly downregulated the HLA-DR expression on CD14^+^ monocytes. Treatment with αIL-10R attenuated IL-10-mediated HLA-DR downregulation in CD14^+^ monocytes while isotype control did not. We also examined whether endogenous IL-10-mediated development of HLA-DR^low/−^ monocytes can be blocked by αIL-10R treatment. We cultured monocytes with supernatant of SuDHL-2 cells in the presence of αIL-10R or isotype control and measured HLA-DR expression on CD14^+^ monocytes. As shown in Figure 5f, in the presence of αIL-10R Ab, an increase in the percentage of HLA-DR^low/−^ monocytes was attenuated when compared with isotype control.

### IL-10-treated monocytes inhibited T-cell activation and proliferation

**Figure 6:**
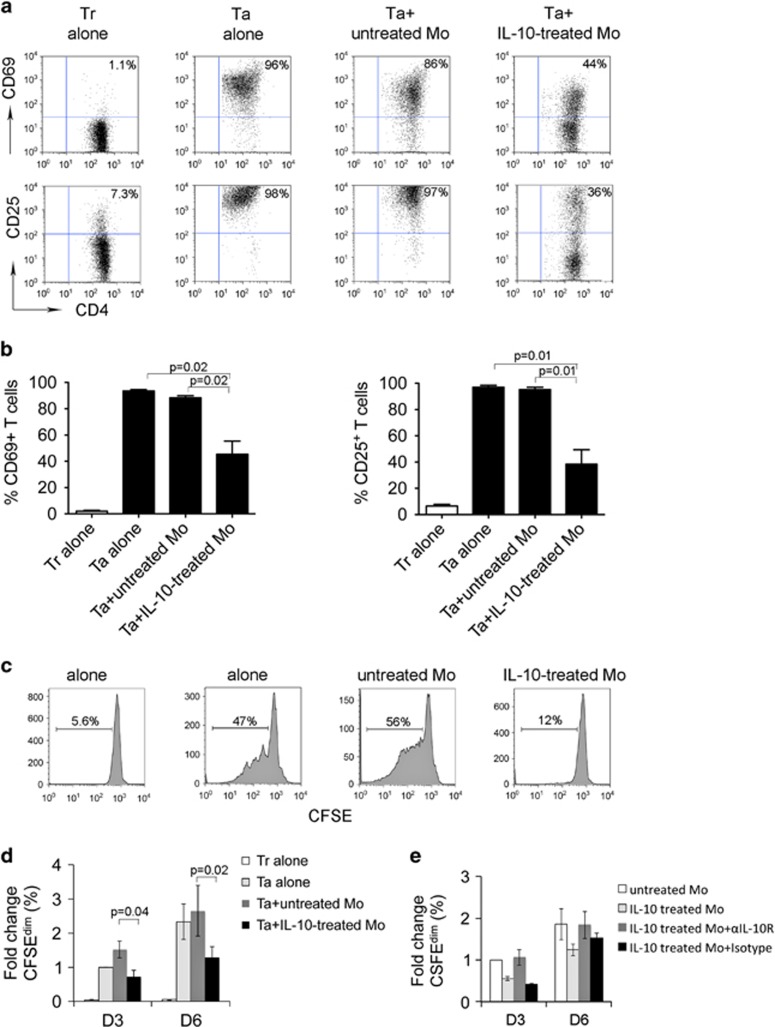
IL-10-treated monocytes inhibit T-cell activation and proliferation. (**a**) Representative plots showing the expression of CD69 and CD25 on activated CD3^+^ T cells (Ta) cocultured with or without untreated or IL-10-treated monocytes (Mo) for 3 days. Expression of CD69 and CD25 on resting T cells (Tr) was measured and used as a control. (**b**) Summarization of CD69 or CD25 induction in Tr or Ta cocultured with or without untreated or IL-10-treated Mo for 3 days, *n*=5. (**c** and **d**) Representative histograms showing proliferation measured by CFSE staining of Tr or Ta cocultured with or without untreated or IL-10-treated Mo for 3 or 6 days. Proliferative capacity was expressed by calculating the number of CFSE^dim^ cells. The results from multiple experiments were summarized in panel (**d**). The percentage change of CFSE^dim^ cells in each group was expressed as fold change when compared with the group Ta alone, *n*=5. (**e**) Graph showing the percentage change of CFSE^dim^ T cells treated with or without untreated or IL-10-treated Mo in the presence of αIL-10R Ab or isotype control for 3 or 6 days, *n*=2.

Next we wanted to test the immune function of IL-10-induced CD4^+^HLA-DR^low/−^ monocytes. To do this, we determined the effect of IL-10-treated monocytes on activation and proliferation of T cells. CD14^+^ monocytes were pretreated with IL-10 for 24 h and then washed three times to remove residual IL-10 prior to coculture with CD4^+^ T cells. Expression of activation markers and proliferation of CD4^+^ T cells were measured by flow cytometry. Compared with resting T cells, activated T cells expressed high levels of CD69 and CD25 and addition of untreated monocytes result in a slight decrease in CD69- and CD25-expressing cells (Figure 6a). In contrast, when activated T cells were cocultured with IL-10-pretreated monocytes, the expression of CD69 and CD25 dramatically decreased (Figures 6a and b).

We then measured the proliferative capacity of CD4^+^ T cells cocultured with IL-10-pretreated monocytes by CFSE staining. As shown in Figure 6c, while activation via OKT3/αCD28 Ab induced significant proliferation indicated by increased numbers of CFSE^dim^ cells, coculture with untreated monocytes slightly enhanced CD4^+^ proliferation. In contrast, we found that IL-10-pretreated monocytes clearly inhibited the proliferation of CD4^+^ T cells when compared with untreated monocytes. The number of CFSE^dim^ T cells was significantly reduced when cocultured with IL-10-pretreated monocytes compared with untreated monocytes (Figure 6d). To determine whether the inhibition of proliferation mediated by IL-10-treated monocytes can be blocked by αIL-10R Ab, we cocultured T cells with untreated or IL-10-treated monocytes in the presence of αIL-10R Ab or isotype control. As shown in Figure 6e, IL-10-treated monocytes strongly inhibited T-cell proliferation. Treatment with αIL-10R attenuated the inhibition of proliferation of T cells cocultured with IL-10-treated monocytes, whereas isotype control did not. On day 6, the effect mediated by αIL-10R Ab was further attenuated compared with that on day 3.

## Discussion

Accumulating evidence shows that the presence of tumor-derived monocytes/macrophages not only suppresses tumor immunity but also supports lymphoma cell growth thereby resulting in a poor outcome in patents with B-cell NHL.^9, 26^ It has been shown that a subset of CD14^+^HLA-DR^low/−^ cells have a crucial role in immune suppression mediated by tumor-derived monocytes in B-cell NHL;^14^ however, the underlying mechanism by which this subset of monocytes is expanded in lymphoma patients is unknown. In the present study, we show that IL-10 is critically involved in the development of the CD14^+^HLA-DR^low/−^ population in B-cell NHL.

It has been found that the frequency of CD14^+^HLA-DR^low/−^ cells is increased in the peripheral blood from cancer patients. CD14^+^HLA-DR^low/−^ cells are increased in several solid tumors, including metastatic melanoma,^27^ hepatocellular carcinoma,^28^ squamous cell carcinoma of the head and neck,^29^ glioblastoma,^30^ prostate cancer^31^ and chronic lymphocytic leukemia.^32, 33^ Our previous study observed^14^ that the numbers of CD14^+^HLA-DR^low/−^ monocytes increased in relapsed B-cell NHL patients. The present study extended this finding by showing that absolute numbers of CD14^+^HLA-DR^low/−^ monocytes are also increased in newly diagnosed B-cell NHL patients and that HLA-DR is downregulated across all three subsets of monocytes. Furthermore, the CD14^+^HLA-DR^low/−^ population of monocytes display suppressive properties and have an impact on patient outcomes in various cancers. For example, increased CD14^+^HLA-DR^low/−^ cells correlate with tumor progression and poor prognosis in hepatocellular carcinoma patients,^34^ and the numbers of CD14^+^HLA-DR^low/−^ cells are associated with extrathoracic metastasis and poor response to chemotherapy in non-small cell lung cancer patients.^35^ In B-cell NHL, we found that patients with increased ratios of CD14^+^HLA-DR^low/−^ monocytes had more aggressive disease and suppressed immune function.^14^ These findings indicate that CD14^+^HLA-DR^low/−^ monocytes are an important contributor to systemic immunosuppression in NHL.^36^

One of the findings in the present study was that the absolute number of total CD14^+^ monocytes was increased in this cohort, which is different to our previous publication in which we reported that there was no difference in the percentage of monocytes between lymphoma patients and controls.^14^ This discrepancy may be due to differing results when the absolute monocyte count and monocyte percentage are used as readouts. Supporting this, other studies from our group have shown that there were different changes in the absolute monocyte count and not in the percentage of monocytes and vice versa for blood phenotype.^30, 37^ Hence, measuring absolute numbers of this cell population might be better than percentages in the context of whole blood.

Little is known about the mechanisms involved in the regulation of HLA-DR on monocytes leading to increased numbers of CD14^+^HLA-DR^low/−^ cells in cancer, especially in lymphoma. In the present study, we have shown that IL-10 is involved in the development of CD14^+^HLA-DR^low/−^ monocytes. We found that IL-10 treatment *in vitro* downregulates HLA-DR expression on CD14^+^ monocytes. Similarly, endogenous IL-10 secreted by lymphoma cells also downregulates HLA-DR expression on CD14^+^ monocytes. Furthermore, we also found that serum IL-10 levels are elevated in patients with B-cell NHL and serum levels correlate with the absolute number of peripheral monocytes. These findings strongly suggest that IL-10 contributes to increased numbers of CD14^+^HLA-DR^low/−^ monocytes in B-cell NHL. We also found that IL-10 regulates the function of CD14^+^HLA-DR^low/−^ monocytes. Supporting the role of IL-10 in facilitating CD14^+^HLA-DR^low/−^ cells in B-cell NHL, we observed that IL-10 pretreated monocytes exhibited suppressive properties by inhibiting activation and proliferation of T cells. This is consistent with our previous finding that CD14^+^HLA-DR^low/−^ cells are the suppressive monocyte phenotype in NHL.

In summary, we show that the absolute numbers of monocytes and percentage of CD14^+^HLA-DR^low/−^ monocytes are increased in newly diagnosed B-cell NHL patients. We observed that serum IL-10 levels are elevated and associated with increased numbers of monocytes. We identified IL-10 as being critically involved in the development of CD14^+^HLA-DR^low/−^ monocytes and find that IL-10 contributes to the expansion of this subset in B-cell NHL. Finally, we found that IL-10 pretreated monocytes exhibit suppressive properties by inhibiting the activation and proliferation of T cells. Taken together, these results reveal a novel role for IL-10 in inducing immunosuppressive CD14^+^HLA-DR^low/−^ monocytes in B-cell NHL that significantly suppress T-cell function. This mechanism could potentially be inhibited to suppress this population of suppressive monocytes.
